# Preparation of Sample Support Films in Transmission Electron Microscopy using a Support Floatation Block

**DOI:** 10.3791/62321

**Published:** 2021-04-08

**Authors:** Natàlia de Martín Garrido, Kailash Ramlaul, Christopher H. S. Aylett

**Affiliations:** 1 Section of Structural & Synthetic Biology, Department of Infectious Diseases, Faculty of Medicine, Imperial College London, South Kensington Campus, Exhibition Road, SW7 2AZ

**Keywords:** TEM, grid preparation, support film, amorphous carbon, graphene, graphene oxide, floatation block

## Abstract

Structure determination by cryo-EM has rapidly grown in the last decade, however sample preparation remains a significant bottleneck. Macromolecular samples are ideally imaged directly from random orientations in a thin layer of vitreous ice. Many samples are refractory to this, however, and protein denaturation at the air-water interface is a common problem. To overcome such issues, support films, including amorphous carbon, graphene, and graphene oxide, can be applied to the grid to provide a surface which sample can populate, reducing the probability of particles experiencing the deleterious effects of the air-water interface. The application of these delicate supports to grids, however, requires careful handling to prevent breakage, airborne contamination, or extensive washing and cleaning steps. We have recently reported the development of an easy-to-use floatation block that facilitates wetted transfer of support films directly to the sample. Use of the block minimises the number of manual handling steps required, preserving the physical integrity of the support film, and the time over which hydrophobic contamination can accrue, ensuring that a thin film of ice can still be generated. In this manuscript we provide step-by-step protocols for the preparation of carbon, graphene, and graphene oxide supports for EM studies.

## Introduction

Over the last decade, breakthroughs, principally in detector technology, but also in other technical fields, have facilitated a succession of substantial increases in the resolution at which biologically relevant systems can be imaged by transmission electron microscopy (TEM)^1,2^. Despite the fact that cryo-electron microscopy (cryo-EM) already allows the resolution of high-resolution structures from as little as 50 μg of protein through single-particle analysis (SPA), cryo-EM sample and grid preparation remain major bottlenecks^3–5^. SPA samples consist of macromolecules distributed approximately randomly within a layer of vitreous ice. The ice must be as thin as possible to maximise the contrast difference between the particles and the solvent.

Biological macromolecules are more stable (i.e. less likely to lose their native structure) in thicker ice, because they remain better solvated. Particles are also often found to be much better distributed over the field of view in ice much thicker than the particle size^6^, and frequently may not be found within holes in the carbon films at all. Additionally, thicker layers of ice decrease the probability of molecules being close to the air-water interface, due to the high surface-to-volume ratio, and it has been estimated that using standard plunge-freezing methods ~90% of particles for cryo-EM studies are adsorbed to the air-water interface^7^. Thicker ice results in undesirably high background, however, due to increased scattering events within the solvent and concomitant attenuation of the signal^6,7^. It is therefore necessary to achieve as thin a layer of vitreous ice as possible; ideally the layer would be only slightly thicker than the particle. The challenge for the researcher, which must be overcome for every different sample applied to a grid, is to prepare specimens thin enough for high-contrast imaging whilst maintaining the structural integrity of the particles within their sample.

Protein adsorption to the air-water interface is accompanied by several, usually deleterious, effects. Firstly, binding of proteins to this hydrophobic interface often induces denaturation of the protein, which proceeds rapidly and is typically irreversible^8,9^; a study conducted using yeast fatty-acid synthase showed that up to 90% of adsorbed particles are denatured^10^. Secondly, evidence from a study comparing the orientation distribution of 80S ribosome datasets collected either on amorphous carbon^11^ or without support^12^, showed that the air-water interface can cause severe preferential orientation compromising 3D reconstruction of the volume^13^. Methods to reduce particle interaction with the air-water interface include supplementation of the freezing buffer with surfactants (such as detergents), the use of support films, affinity-capture or scaffolding of substrates, or accelerated plunging times. The use of surfactants comes with its own problems, as some protein samples may behave non-ideally in their presence, whilst affinity-capturing and scaffolding substrates generally require engineering bespoke grid surfaces and capture strategies. Finally, although there is a lot of research into the development of rapid-plunging devices^14–16^, these require apparatus that is generally not widely available.

Although the standard TEM grid for biological cryo-EM already features a perforated amorphous carbon foil i.e. a Quantifoil^17^, there are a number of protocols available for the generation of additional support films and their transfer to TEM grids. The use of these films is a long-established method for sample stabilisation^18^. Amorphous carbon supports are generated by evaporation and deposition on crystalline mica sheets^19^, from which the layers can be floated onto grids, with the utility of floatation supports as useful tools established in prior reports^20^.

Graphene oxide flakes, typically prepared using a modified version of the Hummers method^21^, have been used as a preferable support structure to amorphous carbon for their decreased background signal as well as the ability to immobilise and stabilise macromolecules^22^. More recently, graphene has seen a resurging interest in its use as a TEM support film due to its mechanical stability, high conductivity, extremely low contribution to background noise^23^, as well as the emergence of reproducible methods for generating macroscopically large areas of monolayer graphene^24^ and transferring it to TEM grids^25^. When compared to amorphous carbon, which undergoes beam-induced motions similarly to, or worse, than ice lacking a support film^11,12,17^, graphene showed a significant reduction in beam-induced motion of cryo-EM images^12^. However, while hydrophilised graphene protected fatty acid synthase from air-water interfacial denaturation, the authors of this study noted that the graphene became contaminated during specimen preparation, likely due to a combination of atmospheric hydrocarbon contamination and from the reagent used to hydrophilise the grids^10^. Indeed, despite many of the superior qualities of graphene, its widespread use is still hindered by derivatisation required to decrease its hydrophobicity^12^, which ultimately is chemically difficult and requires specialist equipment.

This manuscript reports protocols for preparation of amorphous carbon, graphene oxide and graphene sample supports using a 3D-printed sample floatation block^27^ to directly transfer support films from the substrates on which they were generated, to TEM grids. A key advantage of using such a device is the wetted transfer of films, minimising hydrophobic contamination of the supports, thereby minimising the need for further treatment, and reducing the number of potentially damaging manual handling steps. Our approaches are inexpensive to implement and therefore widely accessible and applicable for cryo-EM studies where sample supports are necessary.

## Protocol

1. General preparation of TEM grids pre-support transfer
  1.1 Using a pair of clean, fine tweezers (as preferred by user, we normally use negative-action oblique-tip tweezers), lift and submerge TEM grids sequentially in double-distilled water (ddH_2_O) or ultrapure water, for 10–15 s, followed by ethylacetate, for 10–15 s.
  1.2 Place tweezer, with grid still in grip, to one side to air-dry for ~5 min.
  1.3 Plasma clean the grids to strip the surface of any contaminants accrued through the air or washing steps. We typically plasma clean for 10–15 s in air and with a radiofrequency power of 25 W.
2. General preparation of reagent solutions
  2.1 2% (w/v) uranyl acetate (UAc) solution [CAUTION – UAc: see note below]
    2.1.1 Wrap a 50 mL tube in foil (UAc is light-sensitive and precipitates over time when exposed), fill with 50 mL of ultrapure water and add 1 g of UAc powder.
    2.1.2 Leave the solution stirring for 1 hr to allow for all the UAc to dissolve.
    2.1.3 Store at 4°C.
    2.1.4 Before use, filter 1 mL of stain solution into a small vial using 0.22 μm filter to remove any remaining acetate crystals.
  2.2 Graphene oxide (GrOx) suspension
    2.2.1 Pipette 2.5 μL GrOx into a 1.5 mL tube (1% final concentration).
    2.2.2 Pipette 2.5 μL 10% (w/v) n-dodecyl β-D-maltoside (DDM) detergent into the GrOx and gently mix (0.1% (w/v) final concentration).
    2.2.3 Add 245 μL ultrapure water to the GrOx-DDM mix, and immediately vigorously vortex for 5 min. Use GrOx suspension within 1 hr of preparation, vortex vigorously for at least 1 min before immediate use.
  2.3 10% (w/v) iron(III) chloride (FeCl_3_) solution [CAUTION – FeCl_3_: see note below]
    2.3.1 Carefully weigh 5 g of FeCl_3_ in a weighing boat.
    2.3.2 Transfer to a 100 mL measuring cylinder containing 35 mL ddH_2_O and a magnetic stir bar.
    2.3.3 Plate on a magnetic stirring plate and dissolve FeCl_3_, adding ddH_2_O to a final volume of 50 mL.
    2.3.4 Filter the FeCl_3_ solution through a 0.8 μm syringe filter into a clean bottle for storage.
3. Buffer exchange for carbon support films on mica to prepare negatively stained samples using the support floatation block
  3.1 Wash and plasma-clean TEM grids (we typically use 300 mesh holey-carbon copper grids) as outlined above (Step 1).
  3.2 Pipette between 10–12 μL of sample into the buffer exchange well (with the small channels) of the floatation block, and 10–12 μL of 2% UAc solution (see Step 2.1) for negative staining into the adjacent non-buffer exchange well. NOTE: The well has a volume of 10 μL, however adjust the sample volume so that a convex meniscus is formed at the surface of the liquid to allow proper film floatation. A low volume of sample may cause film breakage.
  3.3 Carefully cut two small pieces of mica with pre-deposited carbon film on top. The mica fragments must be wide enough to fit into the well (3.4 mm width), and longer than the well length (3.45 mm), such that the fragment will sit on the well while carbon is floating and there is enough space to handle the fragment with the tweezers. To handle the carbon we normally use flat negative-action long-tip tweezers.
    NOTE: When cutting the mica fragments, cut using single movements to maintain integrity of the carbon film.
  3.4 Immerse the mica into the well with an approximate angle of 45° until the mica sits on the ramp of the well and a layer of carbon is observed at the surface of the liquid sample.
  3.5 After the initial incubation on sample (typically 20 s–20 min depending on the sample adherence; this requires optimisation by the user), recover the carbon film by withdrawing the mica sheet very slowly to minimise residual viscous sample retention.
  3.6 Carefully blot the mica by tapping the underneath (non-carbon side) with filter paper to remove excess liquid, and subsequently buffer exchange by application to the opposing well (i.e. perform as in Step 2.4), containing the 2% UAc solution. One should appreciate a carbon layer floating on top of the stain solution.
  3.7 Recover the floating carbon layer with the holey carbon-covered side of a washed and plasma cleaned EM grid.
  3.8 Leave grids to air-dry until imaging on a TEM. Ideally, cover the grids during the drying process to avoid airborne contamination.
4. Application of the support floatation block to prepare graphene oxide-coated TEM grids
  4.1 Wash and plasma-clean TEM grids (we typically use 300 mesh holey-carbon copper grids) as outlined above (Step 1).
  4.2 Pipette 10–12 μL of GrOx suspension (see Step 2.2) into the 4 non-buffer exchange wells along the floatation block.
  4.3 Pipette 10–12 μL ddH_2_O or ultrapure water into the remaining 4 buffer exchange wells of the block. This volume of water should be sufficient to form a slight convex meniscus rising above the height of the block.
  4.4 Drop 4 grids gently onto the GrOx suspension of each well for 1 min, holey carbon-covered side contacting the solution.
  4.5 After 1 min, recover each grid carefully by sliding the tweezer into the tweezer groove of each non-buffer exchange well.
  4.6 Very gently and briefly touch the copper, non-carbon-covered side of each grid to the ddH_2_O in the adjacent well. Then carefully and gently hold the grid, water droplet-side down, against a piece of filter paper—blotting off the water will draw the GrOx suspension through the grid by capillary action. NOTE: It is crucial to avoid submerging the grid in the ddH_2_O, so contact should be very brief. When the grid is lifted up, a droplet of water should hold to the underside of the grid. Take care not to move the grid on the filter paper as this could upset the settling of the GrOx flakes.
  4.7 Leave grids in tweezers to air-dry until preparation with sample. Ideally, cover the grids during the drying process to avoid airborne contamination.
5. Application of the support floatation block for the preparation of samples on monolayer-graphene films
  5.1 Wash TEM grids (we typically use 300 mesh holey-carbon gold grids, but other non-copper grids or copper alloy grids are also practicable) as outlined above (Step 1), but omitting plasma cleaning.
  5.2 To deposit grids with graphene we adapted an established protocol for the direct transfer from graphene grown on copper (Cu-graphene) substrates to cryo-EM grids^25^:
    5.2.1 Place four washed grids on top of a Cu-graphene sheet (10 × 10 mm) deposited onto a glass slide and cover each grid with a drop of isopropanol (5–10 μL), thus allowing intimate contact between the monolayer graphene and the grid.
      NOTE: Make sure to place the holey carbon-covered side of the grids in contact with the graphene sheet.
    5.2.2 When the isopropanol is completely evaporated (typically 2 hr), float the Cu-graphene sheet with grids onto 10% (w/v) FeCl_3_ solution (see Step 2.3) in a glass Petri dish and leave to etch at room temperature overnight. Cover the dish to avoid airborne contamination.
    5.2.3 After etching is complete, only the graphene monolayer will remain floating on the FeCl_3_ solution— this should be visible by eye with suitable lighting. Use a loop with diameter larger than the TEM grid size to fish the grids floating on the graphene monolayer and carefully transfer to a glass Petri dish containing ddH_2_O to wash.
      NOTE: Be extremely cautious when fishing the grids to avoid hitting the walls of the Petri dish, which may cause graphene film breakage or bending.
    5.2.4 Repeat wash in water twice more by fishing grids and transferring to a clean Petri dish containing ddH_2_O to remove all residual FeCL_3_.
    5.2.5 Finally, transfer grids into a Petri dish containing sample buffer until sample preparation and plunge-freezing.
      NOTE: The graphene-covered side of the grids must be kept wetted at all times to avoid their exposure to airborne contaminants.
  5.3 Pipette the sample (10–12 μL) into a non-buffer exchange well of the floatation block.
  5.4 When the sample is ready in the block, pick a graphene-coated grid from the buffer solution using a pair of clean tweezers and place onto the surface of the sample-containing well.
  5.5 After an appropriate incubation period (1–5 min depending on the sample; this requires optimisation by the user), pick the grid with a pair of clean freezing tweezers and proceed with blotting and vitrification.

### Caution – UAc

Radioactive and Toxic - Maintain a high level of cleanliness. With the most serious hazard arising from inhalation or ingestion, extra care should be taken to prevent any possibility of inhaling fine particles. Gloves must always be worn when handling or weighing out the uranium salts. Masks and goggles highly recommended. Uranium salts must be disposed of according to the legal requirements set out for radioactive hazards within your state.

### Caution – FeCl_3_

Corrosive and an Irritant - Wash hands and other exposed areas with mild soap and water before eating, drinking or smoking and when leaving work. Provide good ventilation in process area to prevent formation of vapor. Do not breathe mist, vapors, spray. Gloves must always be worn when handling or weighing out the salt. Masks and goggles highly recommended whenever in use.

## Representative Results

**Figure 2:**
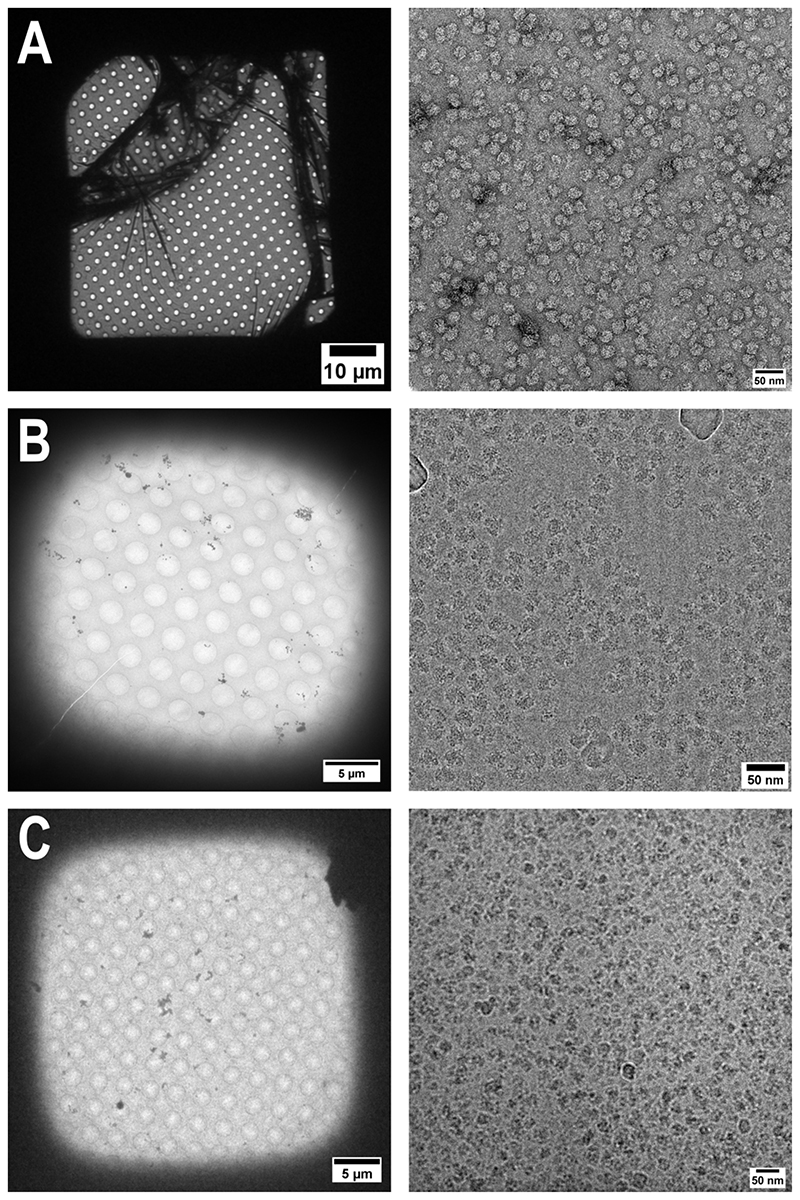
Typical examples of sample support films prepared using the floatation block. Grid square, and image views are shown for amorphous carbon (A), graphene oxide (B) and graphene (C) support films prepared using the floatation block. The amorphous carbon support was used in the preparation of 70S ribosomes for negative staining, whereas the graphene oxide and graphene supports were used in the preparation of 70S ribosomes for cryo-EM. Images in A and C are taken from de Martín Garrido *et al*., 2020 under a Creative Commons (BY 4.0) license.

TEM grids prepared with amorphous carbon supports are typically covered across the entire grid surface. We sometimes see breakage of the carbon film, and frequently see some ruffling (Fig. 2A), but a large number of grid squares are pristine and thus widely applicable for negative staining purposes. The biggest factor affecting the integrity of the support is the carbon thickness, which is determined during carbon evaporation.

Similarly, with our GrOx protocol we routinely achieve good coverage across the entire grid (Fig. 2B). A single application of GrOx suspension for 1 min is sufficient to ensure few areas with multiple layers, which are easy to see due to flake edges. GrOx grids can be prepared quickly from raw materials and are highly protective of the sample, however flake edges, incomplete coverage, and ruffling, are more frequently visible than for the other techniques because of the nature of the GrOx flakes.

Although the integrity of the graphene support film, like the amorphous carbon, depends on the deposition process, areas which are well-covered display the characteristic diffraction pattern of single-layer graphene. Importantly, by keeping graphene support films wetted, we can recover samples from the floatation block after an incubation period and collect data amenable for single particle analysis. This method does not require any other treatment of the graphene for wetting, thereby removing the requirement for expensive equipment to render graphene hydrophilic, and it is best to prepare support films shortly prior to sample preparation and grid freezing (Fig. 2C).

## Discussion

We present protocols for handling of both amorphous carbon and graphene films for cryo-EM sample preparation using a sample floatation block^27^. An STL file for the support block is freely available from the public Thingiverse repository [www.thingiverse.com/thing3440684], and can be 3D-printed with any suitable stereolithography printer from a suitable resin.

The use of carbon films covering a TEM grid usually involves the carbon floatation onto the sample^28^. Our approach to preparing negative stains grids minimises air exposure during support handling, thus reducing contamination and protein denaturation. Preparation of grids using floating carbon in small wells is advantageous to floating a larger surface area i.e. in a water bath or petri dish, in which case mechanical shearing of the carbon occurs much more readily. We note that UAc may be difficult to purchase due to current health and safety regulations at the time of publication, however many other commonly used, non-radioactive, negative staining reagents are available and protocols for their preparation have been described previously^29^. Although we have not used alternative stains with our support floatation block, we do not envisage any differences required for our protocols besides optimisation of incubation time with sample (Step 3.5), which is already inherently sample-dependent.

The key step in our GrOx support preparation protocol is Step 4.6, highlighted by the note to prevent the water and GrOx solution from making contact around the grid edge. Inappropriate mixing of the water and GrOx solutions prevents unidirectional settling of the GrOx flakes by capillary action. Having GrOx flakes on both sides of the carbon foil results in thick layers, thus negating the advantages of using GrOx as a near-single layer support, as well as trapping water between the flakes, which causes contamination of useable areas with additional layers of ice. We note that graphene oxide support preparation is relatively easy to achieve using droplets of solution on Parafilm. However, when performed in that way it is easier to accidentally contaminate the copper side of the grid by mishandling errors, and use of the floatation block reduces the likelihood of this eventuality.

Finally, we present a protocol to prepare graphene covered grids that avoids any kind of graphene pre-treatment to render it hydrophilic, thus reducing its cost and increasing its accessibility. Maintaining a wetted film throughout specimen preparation and applying the sample *in situ* in the block just before freezing is sufficient to allow the generation of suitable ice layers for cryo-EM with a homogeneous sample distribution.

Overall, the protocols presented here minimise sample contact with the air-water interface, therefore reducing sample denaturation and support contamination. For the three support films used in our approaches, we could achieve homogeneous sample distributions across our grids and image intact, well-preserved single particles.

## Figures and Tables

**Figure 1:**
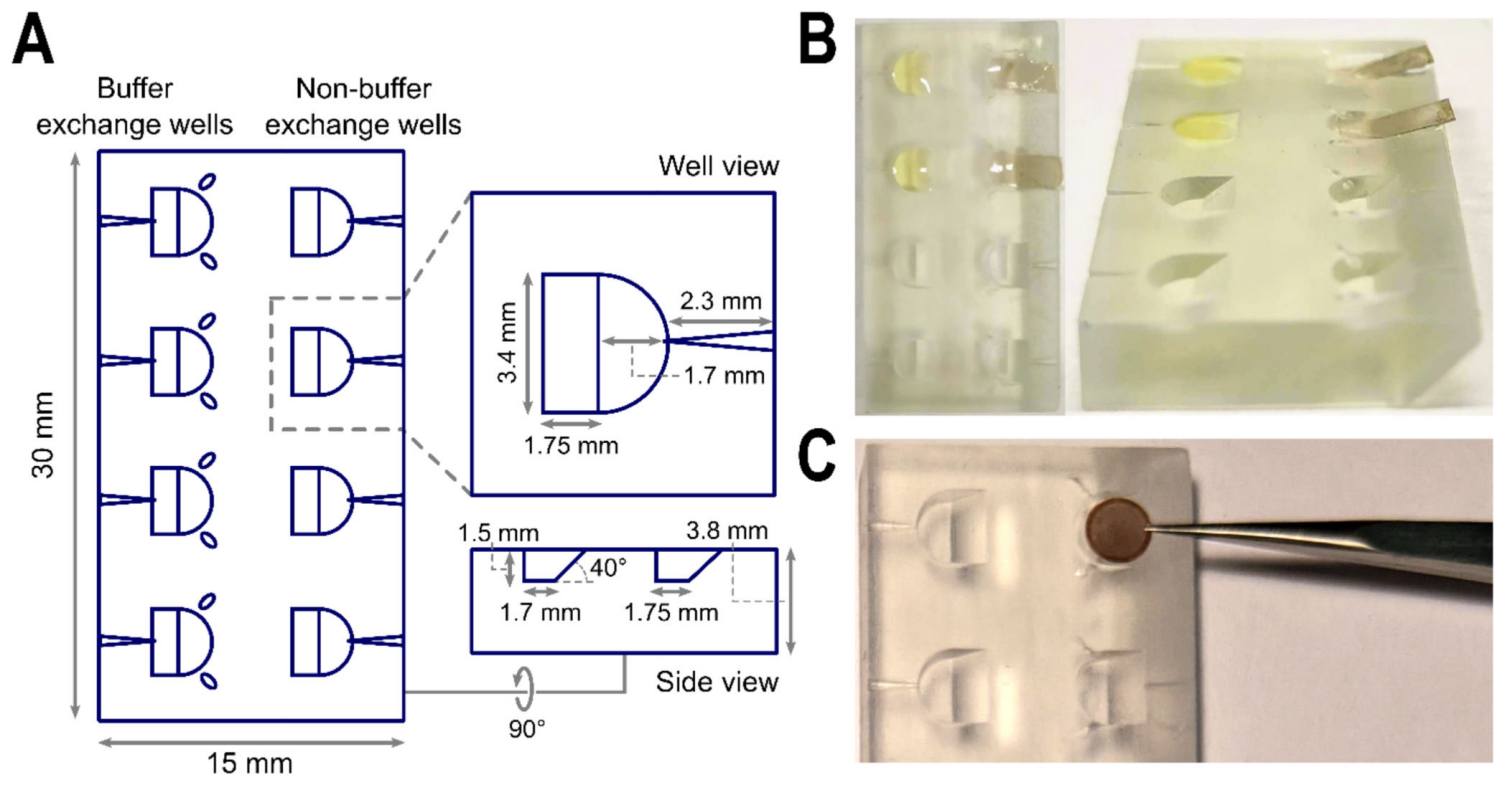
Sample floatation block design and application during support film preparation. A. Schematic of top, well and side views of the floatation block including measurements of the shape, depth and incline. The groove for tweezer tips to rest, as well as channels to insert needles, are indicated. B. Amorphous carbon layers can easily be floated onto the surface of buffer contained within the wells of the floatation block using the ramp, i.e. during the preparation of negatively stained TEM grids. C. The width of the wells is suited to accommodate one TEM grid, whilst the tweezer grooves reduce the need to release and pick up grids unnecessarily during preparation steps, but offer a defined path to recover grids without risk of bending if grids are released. Images in B are taken from de Martín Garrido *et al*., 2020 under a Creative Commons (BY 4.0) license.

## Summary

Sample preparation for cryo-EM is a significant bottleneck in the structure determination workflow of this method. Here, we provide detailed methods for the use of an easy-to-use, 3D-printed block for the preparation of support films to stabilise samples for transmission EM studies.
